# A study on digital inclusion of Chinese rural older adults from a life course perspective

**DOI:** 10.3389/fpubh.2022.974998

**Published:** 2022-09-15

**Authors:** Huan Zhang, Ruimin He

**Affiliations:** ^1^School of Journalism and Communication, Nankai Univerisity, Tianjin, China; ^2^School of Media and Communication, Shanghai Jiao Tong University, Shanghai, China

**Keywords:** smartphone, digital inclusion, digital divide, rural elderly, life course

## Abstract

Digital inclusion is viewed as a crucial strategy for promoting social inclusion and addressing issues related to aging. This study focuses on the digital inclusion practices of rural senior citizens and introduces a life course research perspective to move the study of influencing factors from the proximal to the distal end. The motivations, trajectories, and barriers to digital inclusion among rural elderly groups are presented through participant observation and semi-structured interviews with 34 elderly people in a village in northern China. It was discovered that digital inclusion or exclusion is the cumulative result of life events, social roles, and personal agency in the life course, while it is difficult for the elderly to break away from traditional culture and social relations in rural areas in the digital age. The digital practice is not only an inner adjustment in the process of life stage transition for rural seniors but also an individual pursuit of active aging.

## Introduction

China has 264 million people over the age of 60 by the end of 2020, representing 18.7% of the total population (1). As the population continues to age, policy development and academic research have focused on the issues facing older adults. Among these, the social exclusion and inclusion of the elderly have been major social concerns. With the advancement of information and communication technology, older people face social exclusion in the information age (2). As a result, the concept of digital inclusion is created, emphasizing that “all people should be integrated into the digital trend for personal and social success” (3). The gradual digitization of public services such as daily consumption, medical care, and pensions, as well as daily expenses, makes it imperative for the elderly to adopt new technologies. Some theories, such as post-metaphorical culture, the digital divide, and cultural feedback, as well as specific practices, such as new media workshops and digital back-feeding, have been utilized to assist older individuals in mastering information and communication technology (ICT) and promoting their re-socialization.

With the help of the Digital Village Strategy, the Internet penetration rate in China's rural areas has skyrocketed to 55.9% (1). Based on the author's empirical observation, rural seniors face greater challenges in digital practice than urban seniors due to disparities in economic status, literacy level, social relationships, and life background. However, we know very little about the plight of this more vulnerable group facing changes in information technology. What motivates rural seniors to engage in digital activities? What difficulties and obstacles do they encounter during digital practice? While the majority of recent studies point to age and other objective factors as barriers to digital inclusion, the subjective experience of older people should also be considered. The problem of digital inclusion was not created overnight, and its cumulative nature is emphasized. In light of this, this paper employs a life course perspective to investigate the digital practices of rural seniors and analyzes the key findings.

## Literature review

### From social inclusion to digital inclusion

As the majority of countries and regions gradually enter an aging society, researchers have become aware of several issues that may arise in the survival of the elderly population. Among these issues, the identity change and role adjustment of the elderly group after retirement have garnered considerable attention. In sociology and gerontology, there are two opposing viewpoints on the question of whether or not older people should be socially active. Among them, disengagement theory, also known as social withdrawal theory, proposes that older adults should withdraw from society and return to a peaceful later life as the decline of ability and role loss, whereas activity theory proposes that older adults should actively participate in society to alleviate the emotional depression resulting from the disruption of social roles (4). Fryer (5) coined the term “digital refugees” to refer to older individuals who have subjectively fled or been objectively excluded from the digital world.

Similar to the above-described activity theory, research in the field of communication emphasizes the significance of communication practices in successful aging. Social exclusion, which occurs when individuals or groups are completely or partially excluded from social participation (6), is still unavoidable due to the limitations of the technological environment, social capital, and individual agency. This perspective defines social inclusion as the active promotion of participation opportunities at all levels (7). At the level of the individual, social inclusion is a better adaptation to society and longevity. And for the government, social inclusion is a crucial strategy for addressing the aging population issue.

With a deeper understanding of older adults, some academics have concluded that the term “digital vulnerable group” is a more accurate descriptor, revealing the hope and potential within this demographic (8). In the dual context of a mediated and aging society, digital inclusion can contribute to achieving active aging, maintaining psychological health (9), improving quality of life (10), managing loneliness among older adults (11), enhancing subjective well-being (12). In contrast, older age groups' disconnected behavior and the digital generation gap can have negative consequences. Some researchers are aware that the digital generation gap in Chinese society is exacerbating the digital intergenerational conflict and sparking new conflicts within families and among social groups (13).

The majority of researchers refer to internet use as active aging (14), and argue that the digital inclusion of older people has positive contemporary significance. Active Aging is an updated version of the concept of healthy aging introduced by the World Health Organization in 2002, which aims to improve the quality of life of older people and provide them with the best opportunities for health, participation, and security. Internet use aims to promote the participation and inclusion of older people in society, which is consistent with the objectives of active aging. In response, the Chinese government has elevated “Digital China” to a major strategic decision, with a focus on the construction of smart cities and digital villages to accommodate the social reality that digital technology is being integrated into all areas.

Not only is the learning and use of ICT by older people driven by the objective environment, but it is also viewed as an inherent need for social inclusion and environmental adaptation (15). Although a few scholars emphasize anti-connectivity (16) and advocate for the right to disconnect (17), the majority of studies still uphold a technology-for-good stance, defining lack of internet access or low internet use as a problem to be solved (18). Under this premise, researchers have investigated how to assist older individuals in adopting new information and communication technologies.

### Influence factors for the elderly to use smartphones

The elderly lag behind younger generations in terms of ICT adoption and use (19), and they participate in fewer online activities (20). According to social cognitive theory, older adults' digital inclusion is the result of the interaction between the individual, behavior, and environment (21). Enjoyment to use, expressiveness (22), Self-actualization (23), and connectedness to others (24) among older people themselves facilitate digital practices. Simultaneously, several factors impede the use of smartphones by older people, including both objective and subjective factors. For instance, Lin et al. (25) noted that the phone's Hardware and Software interface caused difficulties in use. According to He and Zhang (26), the digital divide among older adults is caused by a lack of age-appropriate information technology, the neglect of older audiences in online content, the limitations of older adults' characteristics, etc. Additionally, subjective factors, such as reluctance to adopt digital tools (27), lack of confidence (28), and lack of knowledge (29) are also playing a hindering role.

The mainstream literature depicts older individuals as non-users, hostile to technology, and a homogenous population (30). Several qualitative investigations enhance our understanding of the inner lives of older persons. Twenty one digital elders were questioned by (31) about how ICTs influenced their routines and habits. The researchers found that digital seniors have the freedom to determine when and for what purposes ICT use is suitable. Zhou (8) discovered in his study of the adoption of WeChat among older persons that poor demand, limited energy, and technical panic contributed to the non-use or limited usage of new media. This study stressed that subjective needs and perceptions were more influential than objective criteria in the adoption of new media by older persons. In addition to institutional and structural factors, demographic variables and individual psychological and cognitive levels contribute to the digital divide (32). This research also implies that age is not the most critical factor determining digital inclusion in older age groups.

According to Mead (33), our information society is a post-metaphorical civilization in which newer generations teach older generations. Zhou (34) focuses on the pervasive phenomena of cultural feedback in changing societies, and her study demonstrates that cultural feedback has a direct relationship with electronic media. With the intergenerational amplification of the digital gap, Zhou (35) observed that a cultural feedback revolution is occurring within families. Studies on the forms, qualities, challenges, and uses of digital feedback have increasingly proliferated since then (36, 37). Social support is also required for the digitalization of the elderly outside of the home environment. Age-appropriate modifications of digital devices, such as larger fonts, memory-friendly interfaces, and learning interfaces, can accommodate the declining physical functions of older adults (38), and media literacy education can concentrate on developing their engagement and interaction skills (39).

Foremost, Literature on the digital divide indicates that older persons continue to lag in Internet access, digital skills, and digital literacy. These studies shed light on the challenges older persons encounter while using digital technology, but they disregard the impact of interrelated parts of the life course. In addition, the internal heterogeneity and diversity of older persons have been simplified, whilst their subjective experiences and perceptions as users of modern technology are frequently disregarded. In addition, the majority of past research has taken a quantitative approach, which might make it difficult to see the forest for the trees. Therefore, researchers must provide older persons, who are the topic of digital exclusion, with the opportunity to be heard (40). Despite recent qualitative research analyzing the new media use behaviors of older persons in their daily lives (41), which reveals some colorful stories behind the quantitative data, the longer-term and more complicated individual experiences have not yet gotten adequate attention.

Based on this, the study focuses on the front-end variables of digital inclusion behavior, which analyze the effect of the entire life course on the use of digital devices among the elderly and considers the following questions.

*RQ1: What are the distinguishing aspects of smartphone use among rural seniors with distinct life courses?*

*RQ2: What elements in the life courses of an individual inhibit the process of digital inclusion, and what elements promote it?*

## Research design

### Life course: Introduction of a research perspective

In actuality, smartphone adoption is not binary (42), meaning that it does not refer to the option to use or not use. It is a cyclical process in which potential adopters continually seek out new knowledge about a technology, are affected by their peers and family, and adapt their behavior as their life circumstances change (43). Integration into the digital society is a cumulative result of subjective and objective factors, and this paper employs a life course research perspective to investigate the factors impacting smartphone use among rural seniors.

The notion of life Course is retaken from Elder (44), to whom studying the life tracks, involves analyzing the development of human life in its temporal extension and its sociohistorical contexts. The life course of an individual can be found in a set of specific trajectories corresponding to different fields or areas in which the person develops. Such paths may be more or less related, they are presented in a relatively orderly sequence, reflecting positions, transitions, or events experienced by the subject (45). Accumulation and turning points are two fundamental concepts in life course research. Accumulation refers to the positive and negative events, habits, and personalities that result from several early life stages. A turning point is a momentous occurrence that alters or redirects the course of a person's life (46).

As a theoretical and methodological paradigm, it ties age to cumulative strengths or weaknesses, focuses on assessing events and roles in the life course, and analyses a decision, action, or outcome within the context of a person's entire life span. It is frequently used to investigate the interplay between individual experiences and social institutions (47), to explain the diverse outcomes of individual development (48), and to show the elements that influence individual decisions and intentions (49).

In this particular study, social inclusion is a continuous process that unfolds gradually over the life course, and digital inclusion cannot be considered in isolation from the life course. The introduction of the life course perspective has to some extent broadened this type of research, so that the study of digital inclusion and social inclusion of older people is not confined to the description of the old age stage, but also encompasses their entire life history.

People exist initially in their families and then in societies, which determine their normative roles and expected life patterns and provide a framework for comprehending individual behavior and psychology. This study broadly divides the life course of rural elders into three stages: early life, middle life, and late life, to examine the major life events and corresponding social and family roles in each stage and to analyze the cumulative outcomes of the older stages in the context of individual-environment interactions.

**Figure 1:**
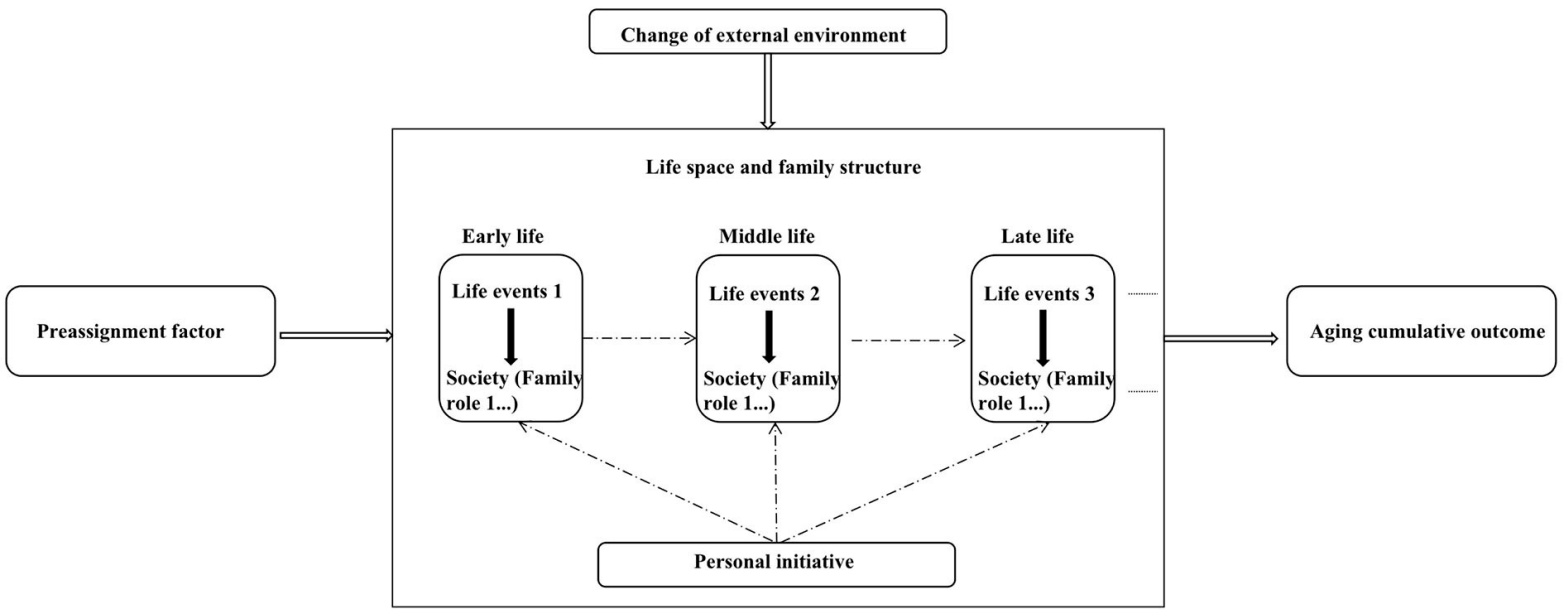
Life course and digital accumulation of rural elderly.

This paper relates to the life course cumulative diagram suggested by Hu (48) and derives the simplified diagram below based on the actual findings of this investigation. Specifically, see (Figure 1). The significance of introducing the life course perspective is threefold: first, to provide a feasible way to understand the behavior, and psychology of the rural elderly; second, to account for the diversity of individuals within the elderly group; and third, to answer the complex reasons for the digital inclusion problem from the perspective of users, and to provide a basis for decision making to advance the digital China strategy.

### Research object and method

The village is situated on a plain 36 km southeast of Tangshan City, Hebei Province. The study site was chosen using a convenience sample based on dialect similarity, geographic proximity, and demographic variables. The village has a total population of more than 800, and the villagers rely on farming as their main source of income. Due to the out-migration of the youthful people and their parents, the phenomena of aging is noticeable in the hamlet, with a large number of elderly and empty-nesters left behind. Due to their low barrier to entry and inexpensive cost, smartphones have become a common Internet access tool for senior citizens in the region. Consequently, this study focuses on smartphone usage and investigates the digital inclusion of rural seniors.

After visiting the area, the researcher recruited study volunteers through radio advertisements and street questions, then grew the study population through snowball sampling. As shown in Table 1, The age distribution of study participants ranged from 60 to 80 years. To investigate the differences within the group and to combine the actual age distribution of the study participants, we referred to the 60 to 66 as the younger-old group, which consisted of 14 individuals, and the 67–73 as the middle-old group, which consisted of 15 individuals, and the 74–80 as the senior-old group, which consisted of 5 individuals.

**Table 1:** Basic information of participants.

**Age group**	**No**	**Age**	**Whether to use smartphones**	**Usage period (year)**	**Number of App usage**
Younger-old	FM2	65	Yes	1	1
group	FM5	66	yes	2	4
	M10	66	yes	1	2
	FM12	60	yes	8	12
	M13	65	yes	2	2
	FM14	63	yes	1	1
	M17	65	yes	0.5	1
	M18	66	no	/	/
	M19	61	yes	5	6
	M22	66	yes	3	3
	M23	66	yes	3.5	4
	M24	60	yes	0.5	2
	FM31	62	yes	5	7
	FM34	63	yes	4.5	5
Middle-old	FM1	72	yes	1	2
group	FM3	73	no	/	/
	FM7	73	yes	1.5	1
	M8	71	yes	6	3
	M9	71	yes	6.5	7
	FM11	69	yes	2	2
	M15	73	yes	3.5	4
	M16	72	yes	1	1
	FM21	72	yes	2	1
	M25	67	yes	1.5	1
	FM26	67	yes	2	2
	FM27	72	yes	1	1
	M28	68	yes	3	4
	M30	68	yes	2	2
	FM33	67	yes	3	2
Senior-old	FM6	75	yes	1	2
group	M20	74	no	/	/
	M29	76	yes	0.5	1
	FM4	76	no	/	/
	FM32	80	No	/	/

FM represents female, M represents male.

Participant observation and semi-structured interviews are the primary research methodologies utilized in this paper. From November 2020 to May 2021, the author observed the smartphone use of the study participants daily to understand their claims, habits, and preferences of using cell phones, including the contents received and disseminated through cell phones, to gain intuitive experiences and understanding, and recorded them. We also employed semi-structured interviews as a data collection technique. In July 2022, we conducted more interviews with four older individuals. At this stage, there were 34 participants, 17 of which were female and 17 of which were male. The retrospective life history study was used to conduct multiple interviews with each elderly individual, and due to the long-time gap, we cross-checked their recollections to present the past life history events, subjective feelings and perceptions, and the social background conditions of the time as objectively as possible.

## Findings

### Smartphone use among rural elderly

People in rural areas are not reliant on the Internet for their employment and daily lives, hence their adoption of the Internet is voluntary and unnecessary. The access order is mostly determined by family structure and economic cost. The village's brief history of the Internet and related equipment access reveals a generational progression of young children, middle-aged parents, and elderly grandparents. Since 2005, rural households have been gradually connected to broadband networks, with the majority of Internet access devices being home PCs and the majority of users being junior and senior high school students. After the development of smartphones, young children were still the first user in the family. After 2012, smartphone ownership began to increase among the middle-aged but had not yet reached the elderly. Only when economic expenses continue to decline will senior citizens have access to the Internet.

The elderly's access to devices is highly dependent on family support. First, the majority of the cell phones used by the elderly are low-end machines costing <1,000 yuan, which are primarily the old cell phones replaced by their children. In addition, most seniors use home broadband, whose operational costs are paid for on a flat-rate basis by their children. And few old people decide to access smartphones on their own. Most are the result of a certain opportunity, coinciding with the replacement of cell phones by young people and operators' promotion in rural areas. And their children have a significant voice in this decision.

Adoption within the senior population reveals some age distinctions. There are five non-smartphone users among the 34 older adults examined in this study, four of whom belong to the senior-old group. Younger people use cell phones for a longer period than middle-olds and senior-olds. However, there is no necessary correlation between the level of usage and access duration. Despite having long access times, some elderly use only a limited number of cell phone features. The levels of literacy, aptitude for learning, and economic status eventually affect how they use Smartphones.

When people reveal their digital practices, it is comparable to unlocking Pandora's Box. Almost to the point of addiction, 17 out of the 29 seniors use smartphones for more than 10 h every day. Because they have more free time, older citizens use their phones more frequently than younger seniors who are still taking care of their grandchildren, or working in the fields.

This study first presents an overview of the actual smartphone usage of the rural elderly and then categorizes them into five categories based on the variation in their usage level to gain a clear picture of their digital inclusion. As (Table 2) shows, The two applications with the highest adoption rates were Kuaishou and WeChat. Of the respondents, 26 used Kuaishou, and 18 used WeChat. It's vital to remember that the levels of digital inclusion vary by age group, with younger and middle-aged groups having higher levels and senior-aged groups having lower levels. The following table displays the specifics.

**Table 2:** Different levels of digital inclusion among rural elderly.

**Inclusion level**	**No**	**Description**
Lower	FM1, FM2, FM7, FM14, M17, M24, M25, FM27, M29, M30	Only can make phone calls, send voice messages *via* WeChat, and browse short videos on Kuaishou.
Low	FM6, M8, M10, FM11, M13, M16, FM21, M22, FM26	Can view videos in Kuaishou, explore WeChat Moments, and take pictures with their phone.
Average	M15, M19, M23, M28, FM33	Can pay with WeChat, find the videos you need on Kuaishou, and use videos to learn how to cook, clean, and do other things.
High	FM5, M9, FM31, FM34	Can shop online, share content on WeChat Moments, upload works to short video sites, and use a few other entertainment apps.
Higher	FM12	Can use their smartphone to interact, make friends, watch videos, buy things, and pay their bills and pensions.

FM represents female, M represents male.

In general, smartphone use among senior people in rural areas is characterized by slow device access, prolonged but shallow use, and a lack of media literacy. They have more psychological and practical impediments while engaging in digital practices. The elements affecting the older group's digital inclusion are continuous and cumulative, making it simple to find in the interviews that they may be linked to an earlier period in their life history. While some elders are fine with the status quo of superficial use, others are highly active users and explorers of smartphones. As will be covered in more detail later, this intra-group variance in use is partially caused by their upbringing and particular experiences.

### Cumulative disadvantage and digital exclusion in the life course of rural elderly

Digital exclusion among older adults is a result of cumulative disadvantage over the life course. The cumulative disadvantage model created by Sampson and Laub (50) showed that breaking the law when you're young increases your chances of becoming delinquent later in life. Cumulative has two meanings in the context of this paper: first, it happens sequentially, affecting people's levels of digital inclusion in later life; and second, there is a chain reaction among them that, taken as a whole, has an effect on the result of digital inclusion.

#### Early in life: Education results initially and lasts a lifetime

The starting point of a person's life course is made up of the family's economic situation, parents' conceptual knowledge, and other variables, which first obviously affect their literacy level and then have an ongoing impact on them throughout their lives. Most residents of the village left school too soon to support their families. The elderly frequently described themselves as illiterate during the interviews.

M15 thought back to his adolescent years when he had to support his grandparents and look for his younger siblings.

“*My father was waiting for me to join the production team so that I could earn work credits because my family was struggling and he wasn't feeling well. I was unaware of the future of education at the time, and only a small percentage of people could pass the exam*.” (M15)

M15 is 73 years old. He was a teenager during the foundation of New China when the country's economy was still comparatively backward and significantly worse in the rural. Many older individuals at his age had similar experiences. As the nation's economy develops, younger-older generations have less financial stress and require less family work while spending a disproportionately longer amount of time in school. However, many rural parents still force their children to leave school at home before completing junior high school because they have a poor understanding of education.

Women have considerably fewer educational possibilities in rural societies where patriarchy is pervasive.

“*The family had a large number of kids at the time, and they were in poverty. I was told as a young girl that studying was unnecessary, so I took care of my sister at home and married young*.” (FM27)

When there is just one smartphone in the home and males are frequently the ones choosing what content to watch, gender discipline and inequity persist into old age.

In addition to restricting the accumulation of wealth and social contact in daily life, a low level of knowledge also prevents later-life resource acquisition and the growth of social networks. Even though older people can access smartphones, they frequently don't know how to utilize them or do so poorly. The digital gap is considered as a natural existence since older people choose to seek young people to perform digital services for them rather than learning how to do so from them.

Access to and use of smartphones are influenced by economic capital and cultural capital. In contrast to M15, M9 has six brothers and a relatively abundant labor force in his family, and he is the youngest, therefore he is not responsible for supporting his family. After graduating from high school, he was assigned to an elementary school, where he taught for more than 30 years. Now that he is retired with a monthly income of almost 4,000 yuan, he purchased a smartphone for himself and his wife earlier. M9 will utilize more of the phone's features than other senior individuals.

Education is a crucial means of class advancement. In the short term, committing oneself to labor output improves the family's economic situation, but in the long run, it pushes them into the resource-poor middle and later life. When it comes to education, the elderly regret,

“*If I had attended school and had a job, I would currently have a pension and could have purchased a cell phone if I so desired. It is inappropriate to request money from children*.” (FM1).

#### Mid-life: The accumulation of role consciousness in rural life restricts personal initiative

In the 1970s and 1980s, along with the reform and opening up process, some daring young people were the first to leave the economically backward countryside and establish their roots in the cities. However, the majority of rural elderly have been in a relatively closed geographical and social environment for a long time, and their social networks are limited to relatives, and acquaintances in the same and neighboring villages. Some older individuals believe that residing in rural areas is the source of backwardness.

“*My city-dwelling cousin had a smartphone approximately ten years ago. I've just been using cell phones for a couple of years, and I'm not even proficient.”* (FM2)

The divide between urban and rural areas is a watershed for digital access. In urban areas, where the Internet was initially developed as an office tool, some people were compelled to become internet immigrants earlier, and the technology then gradually permeated people's daily lives. Farmers engage in manual labor and lack external motivation for digital inclusion. Among interpersonal interactions, villagers are accustomed to group chitchat, square dancing, card games, and other pastimes, forming an offline social way and less expanding social network relationships. Even after accessing the Internet, the number of their WeChat contacts is small, consisting primarily of family members, relatives, residents of the same village, and other acquaintances. In addition, due to their limited economic resources, rural families have developed media consumption habits that prioritize their children, so the brief history of Internet access reveals a sequence of children, parents, and older grandparents. Therefore, the elderly are typically the last members of the family to utilize smartphones.

As an external social structural force, traditional rural life also produces more closed dispositional tendencies, and personal initiative is constrained by the invisible constraints of identity and role. A close-knit society of acquaintances characterizes rural life, where people have a stronger sense of social roles and are easily influenced by the opinions of others.

“*Dressing flamboyantly is imprudent, eating meat every day is a luxury, and we must be especially mindful of our behavior as daughters-in-law.”* (FM1)

They will assume stereotypical roles in accordance with society's general perception. A role is a set of behavioral patterns that people in a particular social position must conform to in order to meet social expectations (51), and it is closely associated with the identity (52). Their professional identity as farmers requires timely field production. In family life, based on their gender identity, women are also expected to perform laborious housework, which affects their ability to accumulate capital in every way. As they grow older, the marginalization of status in the family structure causes a structural exclusion of smartphones.

In conclusion, social structure and personal initiative are intertwined, and the digital inclusion status of rural elders reflects both the limitations of personal initiative due to the role consciousness accumulated in traditional rural life and the behavioral choices made by individuals in the rural environment.

#### Later life: The behavioral discipline of the Familism concept and the absence of intergenerational feedback

Typically, retirement, an increase in leisure time, and the ability to explore new areas of life characterize the third age (53). These characteristics do not, however, apply to the elderly rural population in this study. In contrast, fathers continue to prioritize family interests over personal ones (54), shedding light on the life paths of rural elders. The elderly want to contribute more to the economic development of their families, but because they have no retirement income, many of them continue to perform manual labor even after reaching retirement age. This phenomenon is known as “intergenerational exploitation of the paternal generation by the offspring” (55). And in this regard, rural seniors have a delayed later life.

M26 is a mason, a less labor-intensive occupation that he can still perform at age 67:

“*I earn 120 yuan per day, allowing me to support my son's family of four. When I can no longer do it, my grandchildren will have also grown up and I will be idle to play cards and smartphones.”* (M26)

FM34 further stated, “*I care for my grandchildren and cook for a large family during the day, so I simply do not have time to play on the phone. I only have some free time after 8 p.m to brush kuaishou, WeChat Moments, and Pingduoduo(a discount shopping application).”* (FM34)

At age 70, the rural elderly's life tasks are largely accomplished, and they retire from earning a living and caring for their offspring to enter what Laslett (56) refers to as the “third life” stage, in which they are free to pursue leisure activities without having to worry about household chores and endless caregiving responsibilities. After that, playing mahjong, visiting doors, and using their smartphones become daily activities, and the elderly, who rely on their children for support, make as few financial demands as possible.

In the observed rural families, the process of digital inclusion was not smooth, and feedback between generations was severely lacking. In addition to the objective factor of children leaving home to work or study and not living together for an extended period of time, there are also subjective causes. As the authority established by the traditional power of generation and gender in blood relatives is eroded, older people, particularly men, are inevitably lost, and grandparents do not wish to trouble or burden their children or grandchildren.

“*Who would like to instruct an elderly woman like myself? ”* (FM2) “*I have not made many requests of the children, and they are too busy to instruct me. Moreover, because I am a slow learner, they dislike teaching me.”* (M24)

In addition, the young group has an authoritative role in the digital practice process of the elderly group due to their technological advantages. The young continue to discipline the elderly with words of caution, causing them to view smartphones as a dangerous “black hole” and to refrain from exploring new mobile phone functions.

Current research on digital inclusion focuses on the physiological learning ability of older adults while ignoring their psychological openness to new experiences. Older people will always be mindful of their words and deeds because they have been disciplined by their culture. Even when they have an Internet connection. According to them, the elderly should maintain a certain distance from the Internet, and overactivity is an “anomie” behavior that not only leads to gossip about themselves but also harms their children's reputations.

“*There is an old lady in the village who sings and dances on TikTok, and people laugh at her behind her back.”* (FM1)

Traditional experience will create psychological inertia, resulting in the elderly's digital inclusion being encumbered. The Internet has not entirely broadened the perspectives of the elderly. The majority of elderly individuals view mobile phones as a window to observe society, but not as a means to participate in society.

There are also elderly individuals who have always maintained traditional practices, resisted digital inclusion, and developed a stable system of self-interpretation.

“*The absence of cell phone use has no effect on my life*” (FM4). “*I don't want to learn, it's pointless to learn, and the phone has nothing of value*” (FM3).

Smartphones and the elderly are mutually exclusive, to put it another way. The elderly have not yet broken away from old traditions when confronted with new experiences, forming a social-self, technology-psychological cycle.

When older individuals have Internet access, their decision-making is divided into distinct levels of inclusion in terms of outcomes. The first type, which can be termed the “exclusion type,” consists of senior-aged individuals who have a lower reading level and are more constrained by traditional culture; the second type, which can be termed the “marginal type,” consists of smartphone users who are content with superficial use. The third type, known as the “expectant type,” is eager to engage in additional tasks in order to continuously improve themselves. They are predominantly young-old, have a relatively high reading level, are exposed to more novel experiences, and are more tolerant. In other words, there are age differences among rural elderly smartphone users. The older a person is, the lower their early education level, the less eager they are to learn, and the more likely they are to cling to old habits. In addition to the general cumulative disadvantage, there are factors that stimulate their motivation to access and utilize smartphones, resulting in disparities in the degree of digital inclusion.

### Promotion factors and digital inclusion in the life course of rural elderly

Personality, role adaptation, and unique experiences stimulate personal initiative to varying degrees and, in conjunction with social-technical conditions, contribute to the digital inclusion of the elderly. However, it should be noted that individual agency refers not only to the intentions people have when they act but also to their ability to act in the first place (57). The agency has played a positive role in encouraging the elderly to use digital devices, but it also reveals individual differences in usage. After gaining access to devices, it is still difficult for the elderly to bridge the digital divide due to their cumulative economic and cultural disadvantages.

#### Emotional requirements caused by role transformation

Individual behaviors are not entirely autonomous but are embedded in particular social relationships. In close-knit social networks, the life paths of individuals interact. The analysis of individual behaviors should also consider the people with whom they interact. There are numerous elderly and empty-nesters living in rural areas. After being liberated from their laborious family livelihood and farm work, they felt lost due to life changes and the loss of roles. Some seniors began using smartphones out of boredom and to kill time.

“*Because my children are not at home, they no longer need me, which makes me feel lonely. however, when I play with my phone, time seems to pass quickly, and my loneliness is diminished.”* (FM6) “*After my wife passed away, there is no one talk to, sometimes I can't sleep, I keep playing with my mobile phone, otherwise what can I do.”* (FM7)

The two primary functions of mobile phones, according to Morley (58), are overcoming geographical distance barriers and maintaining social ties. For the elderly, mobile phones offer a new space for communication and emotional compensation, mitigating their sense of age-related alienation and marginalization. The use of smartphones also reflects the adaptation and imagination of elderly people to aging and death,

“*At our age, the more we have to cherish, if we don't contact them now, maybe one day they will die.”* (M16) “*The older I get the more I like to recall, I always want to leave some traces for my children to remember later.”* (M9)

Although many old customs and traditions persist, the desire of the elderly to express themselves through mobile phones is still evident.

In addition, compared to their male counterparts, female seniors are more eager to satisfy emotional needs *via* smartphones. According to the outcomes of digital inclusion, although women have lower levels of literacy, their enthusiasm for playing mobile phone games is significantly greater than that of older men. Zhou's study on WeChat adoption found similar results (8).

### The preservation of personality with age

Personality has a significant impact on older adults' digital inclusion. People who are socially active in their youth will be more active in their later years, whereas introverted people will be less active in their later years, which is a form of cumulative continuity, according to continuity theory. FM12 is a sociable individual who embraces new digital technologies. She spends more than 10 hours per day online, not only perusing online content but also documenting her life on WeChat moments and Kuaishou. The smartphone affords FM12 the opportunity to interact with the outside world, establish a sense of modernity, and achieve psychological inclusion. As the husband of FM12, M19 complains about his wife's conduct,

“*Internet content is accessible to the general public. You should not expose yourself too much. If you live a poor life, you will be ridiculed, while if you live a good life, you will be envious of others*.” (M19)

Although M19 and his wife worked in a large city, he is so introverted that he uses his mobile phone far less than his wife. When FM12 is on a video call with family or children, he simply observes from the sidelines.

While actual society restricts the behavior of the elderly, cyberspace provides a platform for self-expression. Some individuals who are not accustomed to expressing their emotions in person are eager to do so on the Internet. FM5 is one such example.

“*I go out infrequently and do not play mahjong as others do. I enjoy capturing videos of my two cats and uploading them to Kuaishou. I also post photographs of myself with added effects and music. It is a significant record of life.”* (FM5)

From the short videos uploaded by the elderly, it is clear that they are attempting to make themselves appear younger than they actually are. It is not only a reflection of one's life but also a reflection of one's self-worth. According to them, it is worthwhile to record and share these videos, and the likes and comments they receive give them a sense of self.

“*I uploaded my class group photo from my school days on Kuaishou, and I expected others to give me likes and comments, and it was fun*.” (M15, 73 years old)

Although there is an insurmountable gap between the Internet use performance of the elderly and that of the young, the self-presentation of the elderly in the media is a subjective effort to overcome age and role constraints.

#### Unique life events encourage smartphone use

The impact of Turning point on digital inclusion has two sides. For instance, there was a pivotal moment in the lives of the couple FM12 and M19. In the 1980s and 1990s, rapid urbanization occurred in China as a result of reform and opening up. They felt uneasy about the village's poverty and backwardness, so they moved to Beijing and opened a pork shop in a supermarket. In 2007, they returned to their hometown because their children were ineligible to take the college entrance exam in Beijing. After living in a large city, FM12 is extremely open to new experiences. She is adept at utilizing mobile phone features such as instant messaging, moments, watching the news, shopping, and reading novels. According to her own words, she has a strong sense of social progression. Around the age of 40, M8 began a successful career in the animal feed retail industry. The business was thriving. The 2008 melamine incident altered his life trajectory and influenced the evolution of the village's dairy farming methods. For centralized management, the original free-range dairy cows were brought to the cattle farm. As a result, the cattle farm entered into a partnership with a large feed supplier and retailer, and his business suddenly declined. At the age of 60, following a cerebral thrombosis, he became a complete slacker. The enormous psychological gap made him reluctant to leave the house and compelled him to isolate himself. The mobile phone has become an additional means of communication with society.

“*I use my mobile phone to listen to novels and read a wide range of Kuaishou videos. When I do not know how to operate it, my wife will help me.”* (M8)

Certain events can serve as a turning point in an individual's life course. People can make decisions of their own accord the majority of the time, but they are sometimes compelled to act. In a mediated society, it is a practical necessity for some elderly individuals who wish to disconnect to learn how to use digital devices. During the COVID-19 pandemic, numerous senior citizens were required to use smartphones.

“*My grandson was at home during this time for online classes, and it was my responsibility to take pictures of his completed homework assignments, upload them, and sign in on time using my smartphone and tablet. I was the only adult in the home, so I had to learn how to use my phone.”* (FM14)

Similar circumstances convince the elderly who had no intention of adopting the Internet to do so. In some rural households, smartphones are also used as playthings by children. The elderly have learned to show their grandchildren cartoons and short videos on their mobile phones, which has relieved them of the burden of creating games and telling stories.

Personal initiative and life trajectory are intertwined, which encourages smartphone use in later life. The affordances of smartphones provide a remedy for anxiety, and the elderly are reestablishing new ways of life and meaning. As an extension of the body, smartphones create a new spiritual space for the elderly from the standpoints of utility and satisfaction. Some elderly people have gradually developed a positive aging mindset through exposure to the world and interaction with society. The positive experience of media use results in continued use, which assists the elderly in completing the transformation of their social and family roles.

## Conclusion and discussion

The older groups of interest in this study have essentially consistent life paths, and as a result, their digital behaviors share traits like late access, skimpy use, and low literacy. Older rural residents who have worked in agriculture for a long period participate in a small number of social activities. The Internet and contemporary media were not a part of their previous lives. Once they reach senior status, they can just use smartphones. In the tension of life stage transition, factors such as their own personality, life encounters, and spiritual needs stimulate personal initiative to varying degrees. As a result, the willingness and degree of digital inclusion among the elderly vary by exclusion type, marginal type, and expectant type.

**Figure 2:**
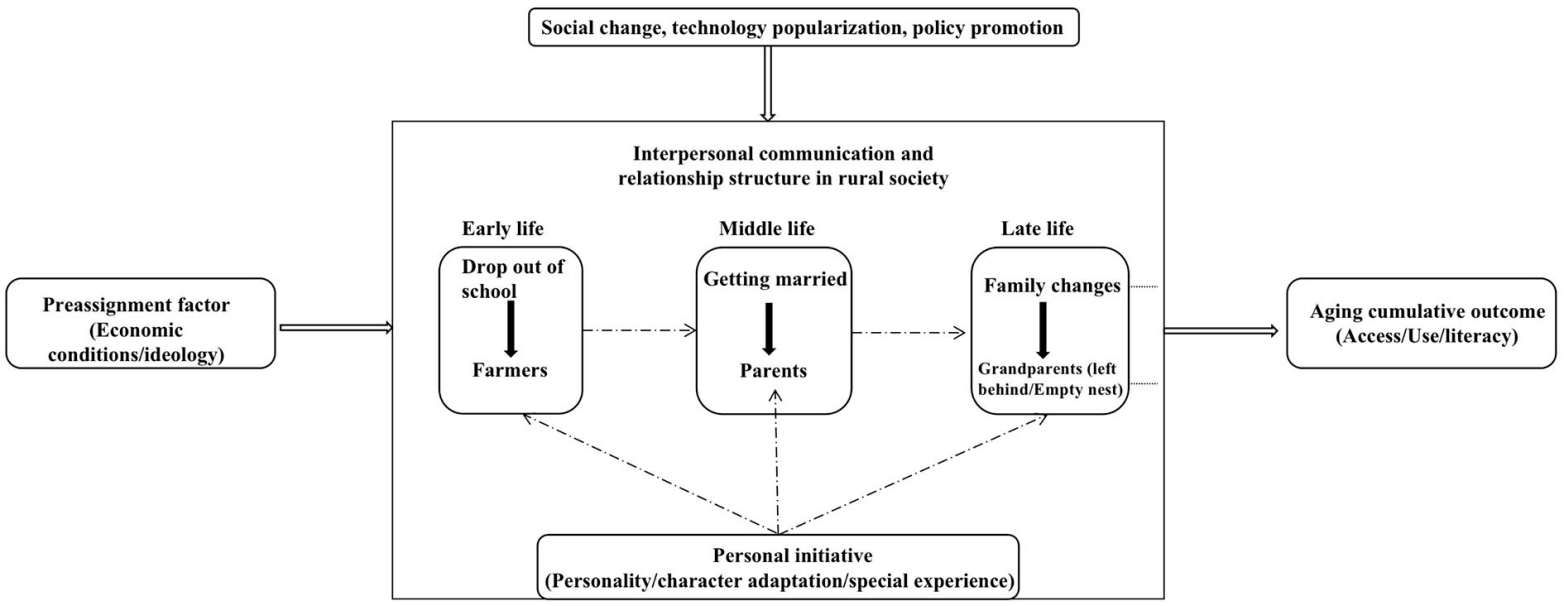
Association between life course and digital inclusion outcomes of rural elderly.

The digital use of rural seniors is not only influenced by the external social environment but also constrained by their subjective life experiences. The life course provides a framework for comprehending individual behavior and psychology by defining the normative roles and expected life patterns of the individual. Digital inclusion or exclusion is the choice of the elderly, which is determined by their experience, concept, mentality, and understanding of life stage, demonstrating a process of accumulation and differentiation. Under the social and cultural norms of rural areas, which are constrained by factors such as knowledge level, economic ability, social interaction, and family structure, the elderly have fallen behind in the use of smartphones, and individual initiative has also promoted their digital practice. The formation procedure is depicted in Figure 2.

The specific elaboration is as follows.

First, ascribed factors (such as economic status, ideology, etc.) brought by the family of origin act on educational opportunities, and then affect the accumulation of other forms of social capital, all of which have an impact on the life course outcome of an individual.

Second, the role consciousness generated by interpersonal communication and relationship structure in rural society affects the elderly's willingness and the degree to accept new things, such as using smartphones.

Thirdly, the accumulation and transition in later life (using or not using smartphones, duration, and degree of usage) is a result of the interaction between people and their environment in a particular time and place and reflects clear individual differences.

Moreover, changes in life stages (social roles and identities) and family situations (member separation, family disintegration, etc.) have a significant impact on behavior and psychology in old age, inhibiting or encouraging the release of personal initiative.

Lastly, specific life experiences (higher social mobility, physical and mental shocks, etc.) can influence personal initiative, ultimately, their willingness and level of digital inclusion.

In addition to the technical divide, the cultural divide stands out among the cumulative factors affecting the digital inclusion of the elderly in rural areas. It is difficult for elderly people in rural areas to escape the shackles of traditional culture and social relations, and they are unable to construct and express themselves freely in cyberspace. In addition, the collision between the inherent experience of the elderly and the modern mindset of the digital society has created a cognitive conflict for them, which is the greatest barrier to the elderly's digital inclusion. In other words, for rural seniors, digital inclusion involves not only technology but also culture. In order to strengthen the digital inclusion of the elderly, we must take care of their inner world and prioritize cultural assistance for the rural elderly, in addition to promoting aging-appropriate transformation and skills training.

In conclusion, when considering how to promote the digital inclusion of rural seniors, it is impossible to ignore the long-term influences on their life course. Instead of viewing old age as a decline in life, it should be viewed as a continuous stage of self-development rather than a hierarchical one (59). In addition, it should be noted that the life course of an individual is embedded in a macroscopic social force and social structure, and this paper examines the digital inclusion of the rural elderly by focusing only on the relevant life course factors and rarely on the social process. This is because the social engagement and interaction of the majority of rural elderly are minimal, and there is little correlation between individual life courses and social historical processes and the use of smartphones. This study has limitations due to its reliance on the elderly of a single village as its research subject, and its conclusions only reflect the reality of certain rural locations in northern China, which may differ from economically developed coastal villages in the southeast. Furthermore, this paper is a retrospective of the past based on the present, and the primary source of content is the self-reports of the elderly respondents, which results in an insufficient presentation of their rich and diverse developmental experiences due to their limited expressibility.

## Data availability statement

The original contributions presented in the study are included in the article, further inquiries can be directed to the corresponding author.

## Author contributions

HZ: study design, data collection, data analysis, and paper writing. RH: paper writing and manuscript review. All authors contributed to the article and approved the submitted version.

## Conflict of interest

The authors declare that the research was conducted in the absence of any commercial or financial relationships that could be construed as a potential conflict of interest.

## Publisher's note

All claims expressed in this article are solely those of the authors and do not necessarily represent those of their affiliated organizations, or those of the publisher, the editors and the reviewers. Any product that may be evaluated in this article, or claim that may be made by its manufacturer, is not guaranteed or endorsed by the publisher.
